# DNA barcoding identification of grafted Semen Ziziphi Spinosae and transcriptome study of wild Semen Ziziphi Spinosae

**DOI:** 10.1371/journal.pone.0294944

**Published:** 2023-12-01

**Authors:** Meng Wu, Haochuan Guo, Mengwei Zhao, Yuping Yan, Yuguan Zheng, Huigai Sun, Donglai Ma

**Affiliations:** 1 School of Pharmacy, Hebei University of Chinese Medicine, Shijiazhuang, Hebei, China; 2 Traditional Chinese Medicine Processing Technology Innovation Centre of Hebei Province, Shijiazhuang, Hebei, China; 3 International Joint Research Center on Resource Utilization and Quality Evaluation of Traditional Chinese Medicine of Hebei Province, Shijiazhuang, Hebei, China; Foshan University, CHINA

## Abstract

Semen Ziziphi Spinosae (SZS) is the dried and ripe seeds of *Ziziphus jujuba* var. *spinosa*. Currently, the yield of naturally grown SZS is unstable owing to environmental factors. Grafting high-quality sour jujube scions onto sour jujube or jujube tree stocks can result in a greater yield. However, the effects of grafting on the quality and gene expression of SZS have rarely been reported. This study used a DNA barcoding technique, high-performance liquid phase-evaporative luminescence detector (HPLC-ELSD), and transcriptomics to investigate the origin and genetic differences between grafted and wild jujube seeds. DNA barcoding identified all samples as *Ziziphus jujuba* var. *spinosa*. HPLC-ELSD analysis revealed a higher content of grafted SZS compared to that of the wild SZS. Transcriptome analysis of the metabolic pathways in SZS showed that 22 and 19 differentially expressed gene sequences encoded enzymes related to flavonoids and saponin synthesis, respectively. Weighted correlation network analysis (WGCNA) identified 15 core genes governing the differences in medicinal components between grafted and wild SZS. This study demonstrated the use of DNA barcoding and fingerprint methods to identify jujube seed species and effectively capture ingredient information of medicinal materials. Additionally, transcriptome technology provided data for identifying core differential genes, facilitating studies on quality differences between grafted and wild SZS.

## Introduction

Semen Ziziphi Spinosae (SZS) was first mentioned in the Shennong Herbal Scripture for its heart-nourishing and liver-detoxifying effects, as well as its ability to promote tranquility and calmness, earning it the nickname “oriental sleeping fruit” [1]. The rapid pace of modern life, increased work pressure, and irregular sleep patterns have led to an increase in insomnia and significantly impacted people’s daily lives [2]. SZS is commonly used to improve sleep [3]. Modern research has demonstrated that SZS contains flavonoids, triterpenoid saponins, alkaloids, fatty acids, and other compounds [4–7], which exhibit certain effects on preventing and treating insomnia. Because SZS is predominantly found in the wild, factors such as climate, terrain, and soil conditions contribute to variations in SZS quality [8, 9]. Consequently, the yield of high-quality SZS has declined continuously, leading to escalating prices.

Grafting connects the rootstock to the scion and involves cutting cells at the incision site and allowing new tissues to merge, forming new plants [10–12]. This technique is crucial for fruit tree breeding because it directly affects the growth, yield, and quality of various fruit trees, such as *Prunus armeniaca* L. [13], plum trees [14], and apple trees [15]. Jujube grafting has been widely studied in China. For instance, *Ziziphus jujuba* Mill. and *Z*. *jujuba* Mill. var. *spinosa* (Bunge) Hu, ex H. F. Chow. Fam. are commonly used in mass production. However, existing research has primarily focused on investigating the morphology, economic benefits, and yield of dates, with limited attention given to the roots and quality following the grafting of *Z*. *jujuba* var. *spinosa*.

With rapid advancements in molecular biology, DNA barcoding has been widely utilized for identifying medicinal plants. The ITS2 sequence, known for its short length, ease of amplification, significant variation, strong identification capabilities, and minimal material requirements, has successfully been applied to identify various traditional Chinese medicines, including *Gnaphalium affine* [16], *Kochia scoparia* [17], *Herbaceous peony* [18], *Rhubarb* [19], and many others [20, 21]. High-throughput sequencing technology has emerged as a valuable method for transcriptomic research [22]. In recent years, this technology has been extensively used in studies on *Pinellia ternata* [23], *Atractylodes macrocephala* [24], *Platycodon grandiflorus* [25, 26], and *Saposhnikovia divaricata* [27], enabling investigations into the synthesis of secondary metabolites in medicinal plants owing to its advantages of high-throughput, rapid processing, and cost-effectiveness.

In this study, DNA barcoding technology was used in conjunction with high-performance liquid chromatography-evaporative light scattering detection (HPLC-ELSD) to analyze the origin and quality of the grafted SZS. The RNA-seq method was used to investigate gene expression differences between grafted and wild SZS, while weighted gene co-expression network analysis (WGCNA) was utilized to explore the core genes associated with the Chinese medicine components of SZS. The findings presented in this study offer valuable data for further examination of the quality disparity between grafted and wild SZS.

## Methods

### Sample collection

The materials used in this study for grafting the date nuts are listed in Table 1. Professor Yuping Yan of the Hebei University of Chinese Medicine identified all samples as mature *Z*. *jujuba* Mill. var. *spinosa* (Bunge) Hu ex H. F. Chou’s dried mature seeds.

**Table 1:** Sample information of SZS.

NO.	Location	Rootstock type	Scion
S1	Baijiazhuang, Hebei	-	-
S2	Cangzhou, Hebei	Gold Jujube	Sour jujube
S3	Fuping, Hebei	Jujube	Sour jujube
S4	Liaoning	Sour jujube	Sour jujube
S5	Shanxi	Sour jujube	Sour jujube
S6	Shanxi	Huping jujube	Sour jujube
S7	Xingtai, Hebei	Sour jujube	Sour jujube
S8	Xindu, Hebei	Sour jujube	Sour jujube
S9	Xinjiang	Jujube	Sour jujube

### Observation of characteristics of SZS

Vernier calipers were used to measure the longitudinal diameter, transverse diameter, and thickness of both the SZS nucleus and SZS. The Keyence digital imaging system (VHX-6000) was used to capture images of the sample shapes.

### Establishment of DNA barcoding

DNA was extracted from the nine samples listed in Table 1 using the Ezup Spin Column Plant Genomic DNA Purification Kit (B518261, Sangon Biotech (Shanghai) Co., Ltd.). The ITS2 sequences were amplified using primers ITS2F 5’-ATGCGATACTTGGTGTGAAT-3’ and ITS3R 5’-GACGCTTCTCCAGACTACAAT-3’, and sequencing was performed by Sangon Biotech (http://sangon.com). After sequencing, the samples were subjected to molecular identification using BLAST (https://blast.ncbi.nlm.nih.gov/Blast.cgi). Interspecific Kimura2-parameter (K2P) genetic distances and neighbor-joining (NJ) phylogenetic trees were constructed using MEGA (Version 3.7.1) software.

### HPLC-ELSD detection

To obtain an accurate SZS powder identification, 1.0 g of the powder was combined with a moderate amount of oil ether. The mixture was incubated for 4 h to facilitate the removal of grease. The sample was subsequently placed in a well-ventilated area to air-dry, and 20 mL of 70% ethanol was added. The solution was heated and refluxed for 2 h, after which it was filtered. The resulting solution was dissolved in methanol, and the volume was adjusted to 5 mL. Spinosin, jujuboside A, and jujuboside B were separately placed into 10 mL volumetric bottles and combined with a constant volume of methanol, resulting in a mixture with concentrations of spinosin at 371 μg·mL^-1^, jujuboside A at 248 μg·mL^-1^, and jujuboside B at 191 μg·mL^-1^.

The concentrations of spinosin, jujuboside A, and jujuboside B in the grafted and wild SZS were assessed using the HPLC-ELSD method, and HPLC fingerprints were established. The chromatographic conditions are listed in S1 Table. Similarity analysis (SA), hierarchical clustering analysis (HCA), principal component analysis (PCA), and orthogonal partial least squares analysis (OPLS-DA) were performed to examine variations in the medicinal component content between the grafted and wild SZS samples.

### Transcriptome analysis

Nine samples from Table 1 were selected, and S1, representing wild SZS, served as the control. RNA of the samples was extracted using the FastPure Plant Total RNA Isolation Kit (RC401-01, Nanjing Vazyme Biotech Co., Ltd.). Transcriptome sequencing was performed using the Illumina NovaSeq 6000 high-throughput sequencing platform (Illumina). Subsequently, Trinity (https://github.com/trinityrnaseq/trinityrnaseq) and BUSCO (https://busco.ezlab.org/) software were used to splice and assemble clean reads after removing original junctions and low-quality reads, resulting in transcripts of varying sizes and unigenes. The reconstructed transcripts were aligned against relevant databases (Swiss-Port, NR, Pfam, Go, EggNOG, and KEGG) using the BLAST+ (version 2.9.0) software to obtain information on unigenes and transcript annotations. Statistical analyses of annotations were performed. Differentially expressed genes associated with flavonoid and saponin biosynthesis in SZS were identified, and an expression pattern heat map of the differentially expressed unigenes was generated using R software.

### Weighted gene co-expression network (WGCNA) analysis

The WGCNA is a method that allows the identification of modules and the exploration of cooperative changes within gene sets, thereby facilitating the study of gene interactions at a global level [28]. WGCNA was constructed using the WGCNA package in R (version 4.0.3) to analyze the correlation between genes and phenotypes, specifically the levels of spinosin, jujuboside A, and jujuboside B. The module identification process involved the following parameters: soft power of 12, minimum module size of 30, minimum kME (module eigengene connectivity) threshold of 0.3, and merge cut height of 0.25. The resulting gene modules were visually analyzed using Cytoscape 3.5.1.

### q-RT PCR analysis

RNA was extracted from nine samples using the FastPure Plant Total RNA Isolation Kit (RC401-01, Nanjing Vazyme Biotech Co., Ltd.). ChanQ Universal SYBR qPCR Master Mix (Q711-02, Nanjing Vazyme Biotech Co., Ltd.) was used to reverse transcribe the extracted RNA. Primer premier (version 5.0) software was used for primer design, and amplification and detection were performed using HiScript Ⅲ RT SuperMix for qPCR (+gDNA wiper) (R323-01, Nanjing Vazyme Biotech Co., Ltd.) and a Mastercycler® nexus PCR (Eppendorf). Fifteen highly correlated genes were selected for verification using quantitative real-time polymerase chain reaction (qRT-PCR), with *Actin 8* serving as an internal reference. Primer sequences for the selected genes are listed in S2 Table.

## Results

### Morphological observation of samples

**Fig 1:**
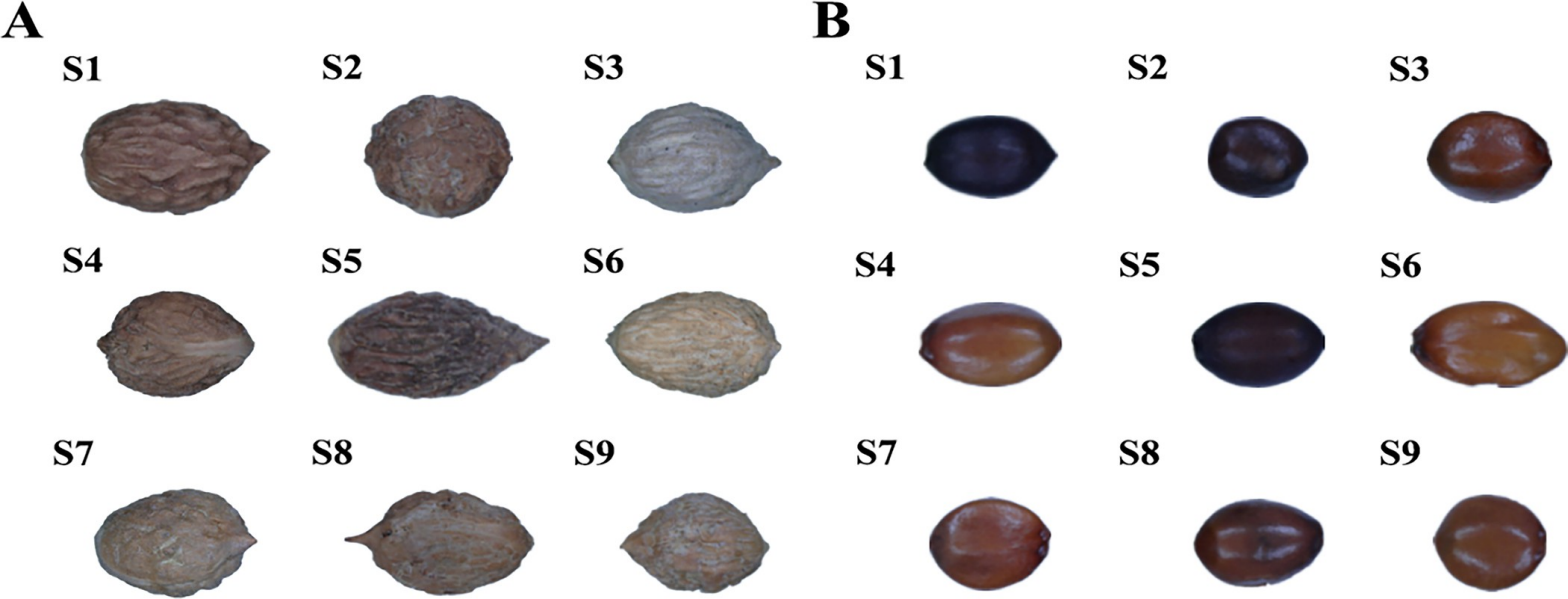
The apparent morphology of Semen Ziziphi Spinosae (SZS) samples. (A) SZS date pits. (B) SZS.

The apparent morphologies of the nine samples are shown in Fig 1. There were evident differences in the characteristics of the various sour jujube components (date pits and kernels). As shown in S3 Table, the longitudinal diameter of the stones ranged from 0.97 to 1.63 cm, the transverse diameter of the seeds ranged from 0.67 to 0.98 cm, and the thickness of the seeds ranged from 0.58 to 1.03 cm. Most date pits were oval-shaped, whereas S2 exhibited a slightly rounded shape (Fig 1A). The SZS (sour jujube kernels) had a longitudinal diameter ranging from 0.5 to 0.98 cm, a transverse diameter from 0.46 to 0.65 cm, and benevolence from 0.24 to 0.34 cm. As shown in Fig 1B, SZS seeds were primarily oval, mostly of them being purple-red. S1, S2, and S5 were purple-brown, whereas a few, such as those of S6, were orange. The morphological disparity between the grafted and wild sour jujubes was substantial, whereas the difference between the SZS was minor.

### Molecular identification using ITS2

The ITS2 sequence length of SZS ranged from 460 bp to 515 bp (S4 Table). The GC content ranged from 60.04% to 60.97%. BLAST similarity retrieval was conducted on the successfully obtained sequences, and all results showed a high degree of matching. This identified *Z*. *jujuba* var. *spinosa* as the species with the highest similarity, confirming the molecular identity of the nine samples.

### NJ trees of grafted and wild SZS

**Fig 2:**
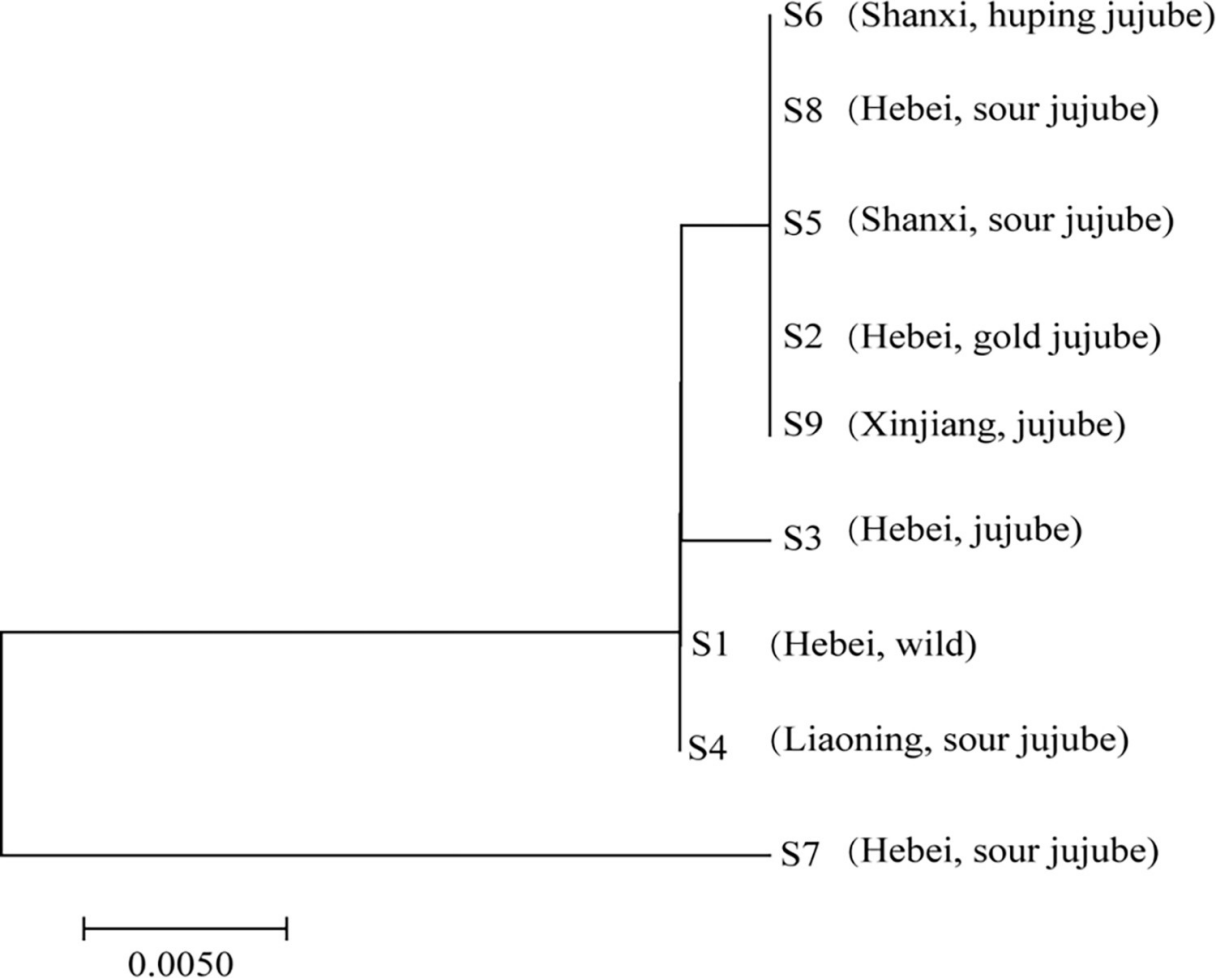
Neighbor-joining (NJ) tree of the nine Semen Ziziphi Spinosae (SZS) samples.

The K2P genetic distance was calculated for the ITS2 sequences of wild and grafted SZS (S5 Table). Smaller values in the table indicate closer genetic relationships. Genetic distance values ranged from 0.000 to 0.038, with an average of 0.005. Using the similarity search and nearest distance methods, an NJ tree was constructed with the bootstrap value set to 1000 repetitions (Fig 2). Notably, the wild sample (S1) was not clearly distinguished from the other graft samples. The results of the NJ tree and K2P genetic distance analyses revealed a close relationship between wild and grafted SZS.

### Contents of medicinal components in SZS

The three control peaks correspond to the individual components of the sample (S1 Fig). The linear regression equations for the standard products are listed in S6 Table. The *r*^2^ values for all three components exceeded 0.9995, confirming a strong linear relationship between each component. The concentrations of the three medicinal ingredients in SZS are presented in S7 Table. Spinosin, jujuboside A, and jujuboside B content ranged from 0.0820 to 0.1412, 0.0386 to 0.0793, and 0.0130 to 0.0409 mg/g, respectively.

### Fingerprint establishment and similarity evaluation

The HPLC-ELSD chromatogram was used in the similarity evaluation system for the chromatographic fingerprinting of TCM (2004 A) and fingerprint establishment (S2 Fig). Through the fingerprints of nine SZS samples, a total of eight common peaks were identified. By comparing the fingerprints of the mixed control products, three peaks were identified: peak 3, spinosin; peak 7, jujuboside A; and peak 8, jujuboside B. The fingerprint similarity evaluation results are presented in S8 Table. The similarity between samples ranged from 0.701 to 0.976. The similarity between S2 and S6 was the lowest, whereas that between S7 and S8 was the highest.

### Difference of medicinal component between wild and grafted SZS

**Fig 3:**
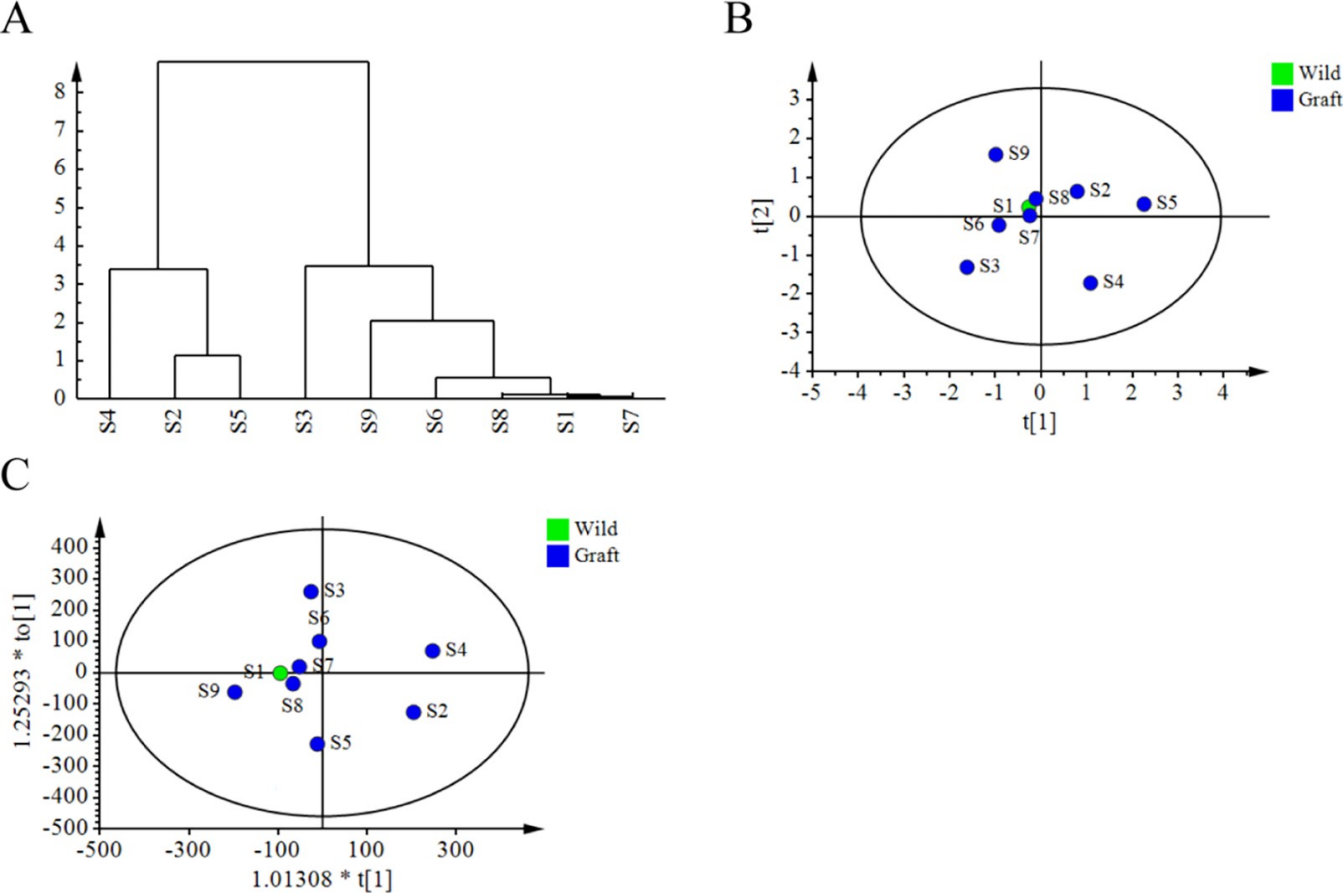
Chemical pattern recognition analysis. (A) Cluster analysis The tree diagram of cluster analysis for Semen Ziziphi Spinosae (SZS). (B) Principal component analysis (PCA) of SZS samples. (C) Orthogonal partial least squares analysis (OPLS-DA) of SZS samples.

The peak areas of spinosin, jujuboside A, and jujuboside B were imported into the SIMCA software for chemical pattern recognition analysis (Fig 3). Cluster analysis revealed that the nine samples could be divided into two categories (Fig 3A). S2, S4, and S5 were grouped into one category, whereas S1, S3, and S6–S9 were grouped into another. The wild sample (S1) clustered together with most of the grafted samples. The PCA and OPLS-DA results indicated no significant differences between the wild and grafted samples (Fig 3B and 3C), suggesting minimal variation in the medicinal ingredients between the two types of samples.

### Transcriptome sequencing data

A total of 195.68 Gb of clean data was obtained through transcriptome analysis of 27 samples. They were assembled using Trinity software, resulting in 94,800 transcripts. From these transcripts, 44,510 unigenes were identified (S3 Fig). Among these, 16,274 (37%) had lengths ranging from 200 to 500 bp, indicating the prevalence of short unigene sequences. There were 11,193 (25%) unigenes with lengths ranging from 501 to 1,000 base pairs (bp). Additionally, there were 4,716 (11%) unigenes with lengths ranging from 1,001 bp to 1,500 bp. The remaining unigenes (12,327 bp) had lengths exceeding 1,500 bp.

### Function annotation

The obtained unigenes were subjected to annotation using various databases, including KEGG, NR, SwissProt, EggNOG, GO, and Pfam. The annotation results revealed that 21,973 unigenes (49.37%) were annotated using GO, 11,855 (26.63%) using KEGG, 213,377 (48.03%) using EggNOG, 26,592 (59.74%) using NR, 19,481 (43.77%) using SwissProt, and 18,339 (41.2%) using Pfam. These annotations provided comments on the unigenes, with the proportion of commented unigenes in each database ranging from 25% to 60%.

### Differential genes expression analysis

Differentially expressed genes (DEGs) may play a significant role in distinguishing jujube varieties. Hence, we identified DEGs between wild and grafted SZS samples, as illustrated in S4 Fig. The number of DEGs varied across different sample groups, ranging from 1,300 to 7,400. The most DEGs were observed between S8 and S1, with 4,575 up-regulated and 2,780 down-regulated genes. In contrast, the smallest number of DEGs was found between S2 and S1, with 434 up-regulated and 874 down-regulated genes. The up-regulated and down-regulated genes between S9 and S1 and between S5 and S1 exhibited consistent patterns.

### KEGG enrichment analysis of DEGs

**Fig 4:**
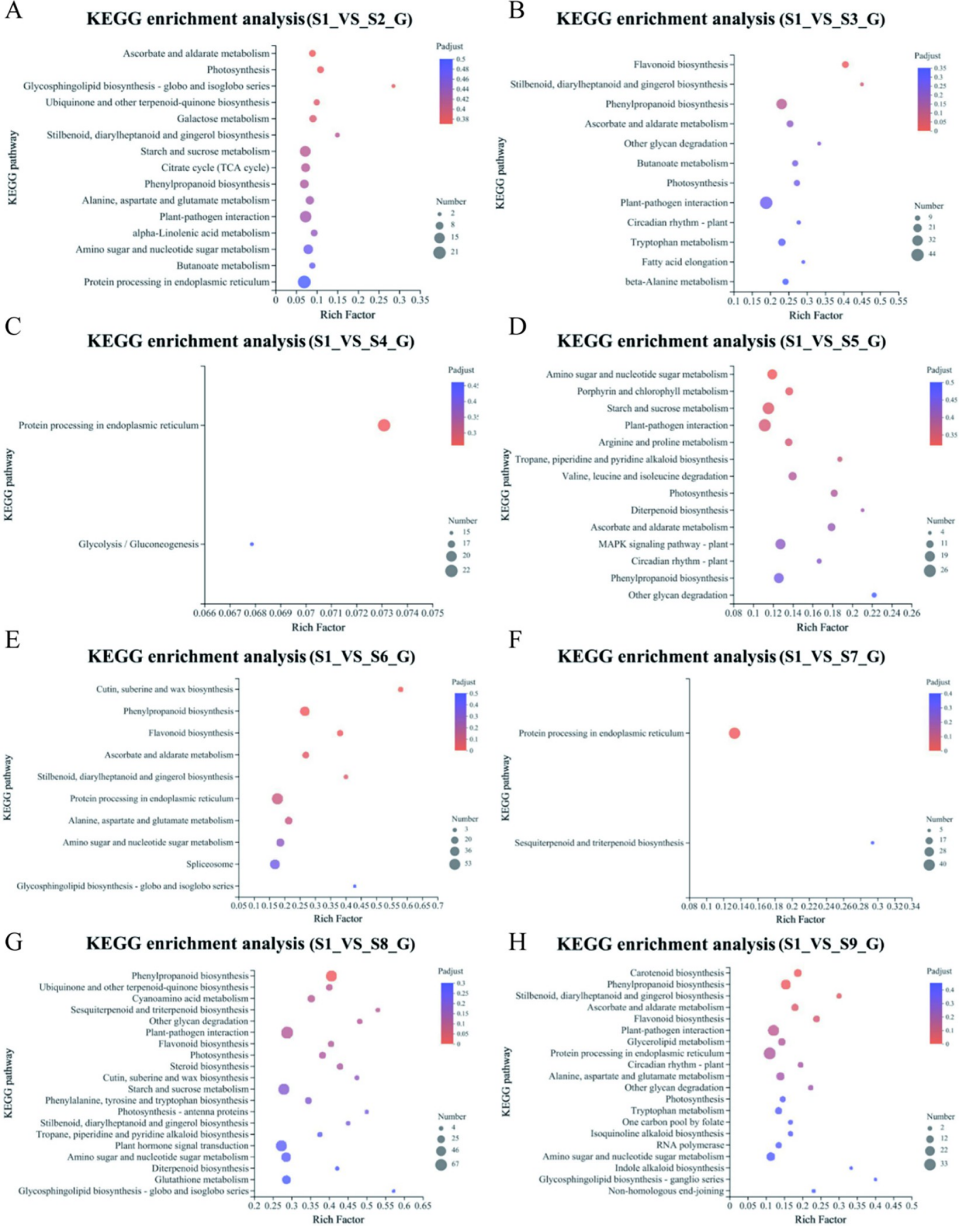
KEGG pathway annotation statistics of grafted and wild Semen Ziziphi Spinosae (SZS) differentially expressed genes (DEGs.). (A) KEGG enrichment analysis of S1 and S2 DEGs. (B) KEGG enrichment analysis of S1 and S3 DEGs. (C) KEGG enrichment analysis of S1 and S4 DEGs. (D) KEGG enrichment analysis of S1 and S5 DEGs. (E) KEGG enrichment analysis of S1 and S6 DEGs. (F) KEGG enrichment analysis of S1 and S7 DEGs. (G) KEGG enrichment analysis of S1 and S8 DEGs. (H) KEGG enrichment analysis of S1 and S9 DEGs.

KEGG enrichment analysis was used to identify the key metabolic pathways or signal transduction pathways involving the DEGs. The transcriptome genes of grafted and wild SZS were subjected to KEGG enrichment analysis, and the top 20 enrichment results are displayed in Fig 4, based on a significance threshold of *P* < 0.5. The 457 DEGs between S2 and S1 were enriched in 104 KEGG metabolic pathways, with the pathway “protein processing in endoplasmic reticulum” exhibiting the highest concentration of DEGs. The 1,449 DEGs between S3 and S1 were enriched in 126 metabolic pathways, with the most abundant pathway being “plant-pathogen interaction.” Between S8 and S1, the 2,167 differential genes were enriched in 130 metabolic pathways, with “plant-pathogen interaction” as the most enriched pathway. The 400 DEGs between S4 and S1 were enriched in 102 metabolic pathways, with “protein processing in the endoplasmic reticulum” being the most enriched pathway. The 823 DEGs between S5 and S1 were enriched in 117 metabolic pathways, with the “plant-pathogen interaction” being the most enriched pathway. Between S6 and S1, the 1,287 DEGs were enriched in 121 metabolic pathways, with “protein processing in the endoplasmic reticulum” being the most enriched pathway. Between S7 and S1, the 770 DEGs were enriched in 117 metabolic pathways, with “protein processing in the endoplasmic reticulum” as the most enriched pathway. Finally, the 825 DEGs between S9 and S1 were enriched in 119 metabolic pathways, with “protein processing in the endoplasmic reticulum” as the most enriched pathway.

### Analysis of genes related to the synthesis of secondary metabolites

#### Expression analysis of genes related to flavonoid biosynthesis pathway

**Fig 5:**
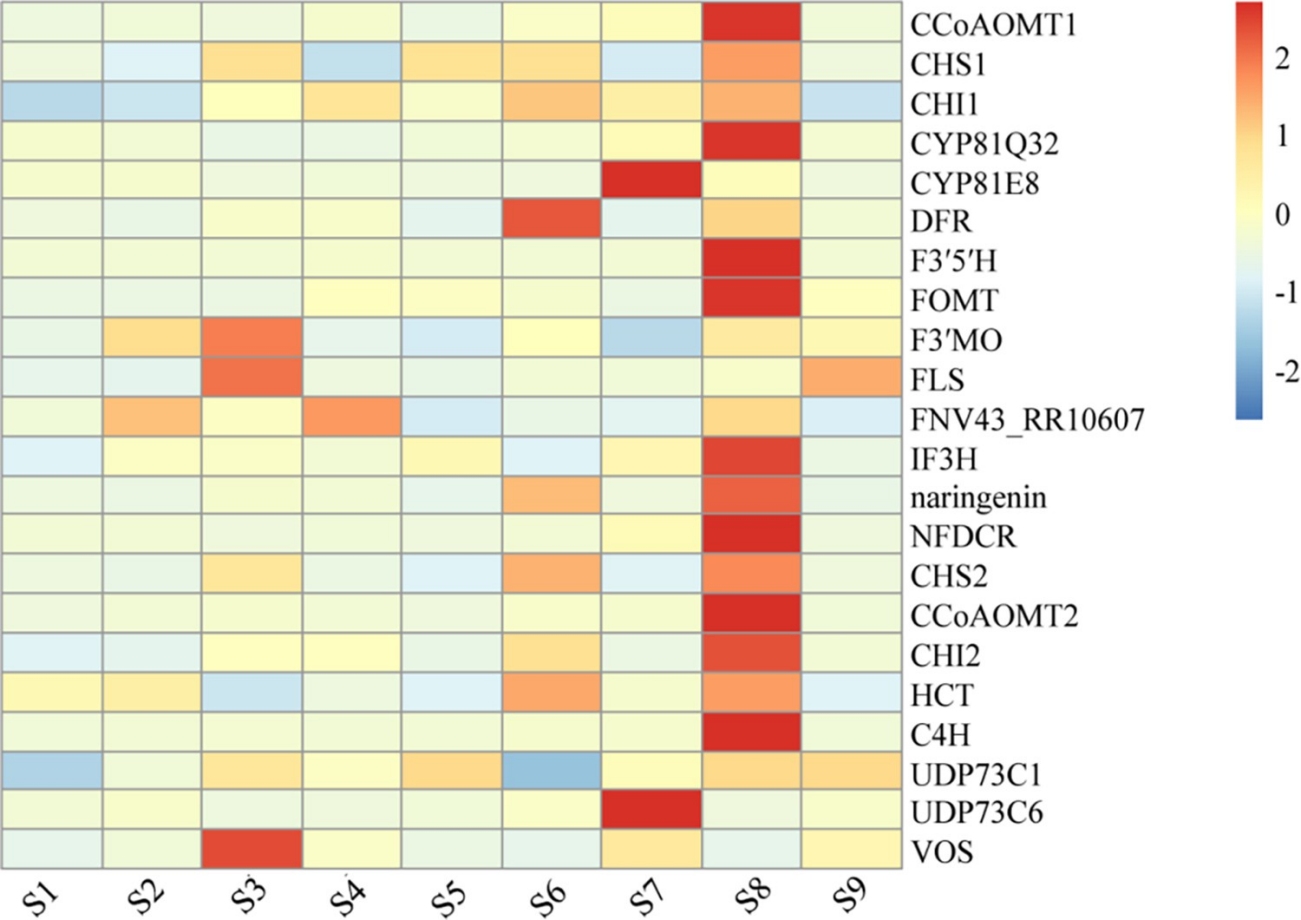
Cluster heat map of differentially expressed genes (DEGs) related to flavonoid synthesis.

By screening genes involved in the flavonoid biosynthesis pathway across the eight sample groups, 22 DEGs were annotated as 19 biosynthetic enzymes, excluding those with low expression levels. An expression cluster analysis of these 22 DEGs was conducted, and the results are shown in Fig 5. Additional genetic information is presented in Table 2. In the wild sample (S1), the differential genes did not exhibit significant up- or down-regulation. However, these genes were either significantly up- or down-regulated in the grafted samples. Specifically, *F3’Mo* and *FNV43_RR10607* were significantly up-regulated in S2, while *F3’MO*, *FLS*, and *VOS* were significantly up-regulated in S3. *CHI* and *FNV43_RR10607* were significantly up-regulated in S4, whereas *CHS* and *UDP73C1* were significantly up-regulated in S5. In S6, *CHI*, *DFR*, naringenin, *CHS*, and *HCT* were significantly up-regulated, while *CYP81E8* and *UDP73C6* were significantly up-regulated in S7. *CCoAOMT*, *CYP81Q32*, *F3’5’H*, *FOMT*, *IF3H*, *naringenin*, *NFDCR*, *CHS*, *CHI*, *HCT*, and *C4H* were significantly up-regulated in S8, and *FLS* was significantly up-regulated in S9.

**Table 2:** Flavonoid synthesis-related genes.

Gene	Abbreviation
caffeoyl-CoA O-methyltransferase	CCoAOMT
chalcone synthase	CHS
chalcone-flavanone isomerase	CHI
cytochrome P450 81Q32	CYP81Q32
cytochrome P450 81E8	CYP81E8
dihydroflavonol 4-reductase	DFR
flavonoid 3’,5’-hydroxylase	F3′5′H
flavonoid 3’,5’-methyltransferase	FOMT
flavonoid 3’-monooxygenase	F3′MO
flavanol synthase	FLS
hypothetical protein FNV43_RR10607	FNV43_RR10607
isoflavone 3’-hydroxylase	IF3H
naringenin	naringenin
non-functional NADPH-dependent codeinone reductase	NFDCR
Shikimate O-hydroxy cinnamoyl transferase	HCT
trans-cinnamate 4-monooxygenase	C4H
UDP-glycosyltransferase 73C1	UDP73C1
UDP-glycosyltransferase 73C6	UDP73C6
vinorine synthase	VOS

#### Expression analysis of genes related to saponins biosynthesis pathway

**Fig 6:**
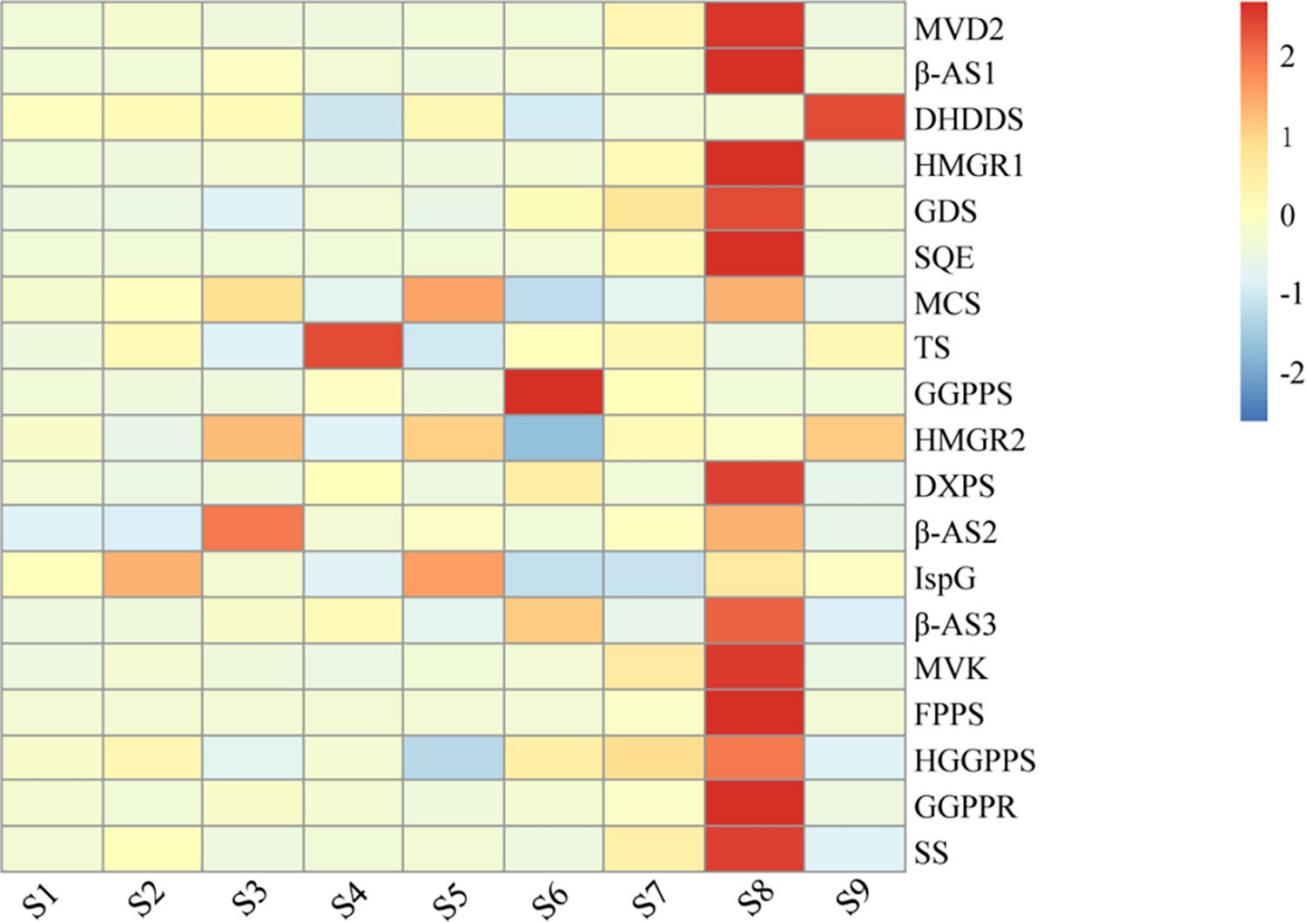
Cluster heat map of differentially expressed genes (DEGs) related to the biosynthesis of saponins.

Jujubosides A and B are triterpenoid saponins. By screening the genes involved in the saponin biosynthesis pathway across the eight groups of samples, 19 DEGs were identified. These DEGs, excluding those with low expression levels, were annotated as 16 biosynthetic enzymes (Table 3). The expression cluster analysis of these DEGs is shown in Fig 6. In the wild sample (S1), the genes did not exhibit significant up- or down-regulation. However, the expression of specific genes was significantly up-regulated in the remaining samples. Notably, *IspG* was significantly up-regulated in S2. In S3, *MCS*, *HMGR*, and *β-AS* were significantly up-regulated. TS was significantly up-regulated in S4. S5 showed significant up-regulation of *MCS*, *HMGR*, and *IspG*. *GGPPS* and *β-AS* were significantly up-regulated in the S6. In S8, *MVD*, *β-AS*, *HMGR*, *GDS*, *SQE*, *DXPS*, *MVK*, *FPPS*, *HGGPPS*, *GGPPR*, and *SS* were significantly up-regulated. Additionally, *DHDDS* and *HMGR* were significantly up-regulated in S9.

**Table 3:** Genes related to the biosynthesis of saponins.

Gene	Abbreviation
(-)-germacrene D synthase	GDS
2-C-methyl-D-erythritol 2,4-cyclodiphosphate synthase	MCS
3-hydroxy-3-methylglutaryl-coenzyme A reductas	HMGR
4-hydroxy-3-methylbut-2-en-1-yl diphosphate synthase	IspG
beta-Amyrin Synthase	β-AS
dehydrodolichyl diphosphate synthase	DHDDS
diphosphomevalonate decarboxylase MVD2	MVD2
farnesyl pyrophosphate synthase	FPPS
geranylgeranyl diphosphate reductase	GGPPR
geranylgeranyl pyrophosphate synthase	GGPPS
heterodimeric geranyl pyrophosphate synthase small subunit	HGPS
mevalonate kinase	MVK
probable 1-deoxy-D-xylulose-5-phosphate synthase	DXS
squalene monooxygenase	SQE
taraxerol synthase	TS
1-deoxy-D-xylulose-5-phosphate synthase	DXPS
squalene synthase	SS

### Co-expression network analysis of differential genes related to medicinal components of wild and grafted Jujube seeds

#### Construction of co-expression module

**Fig 7:**
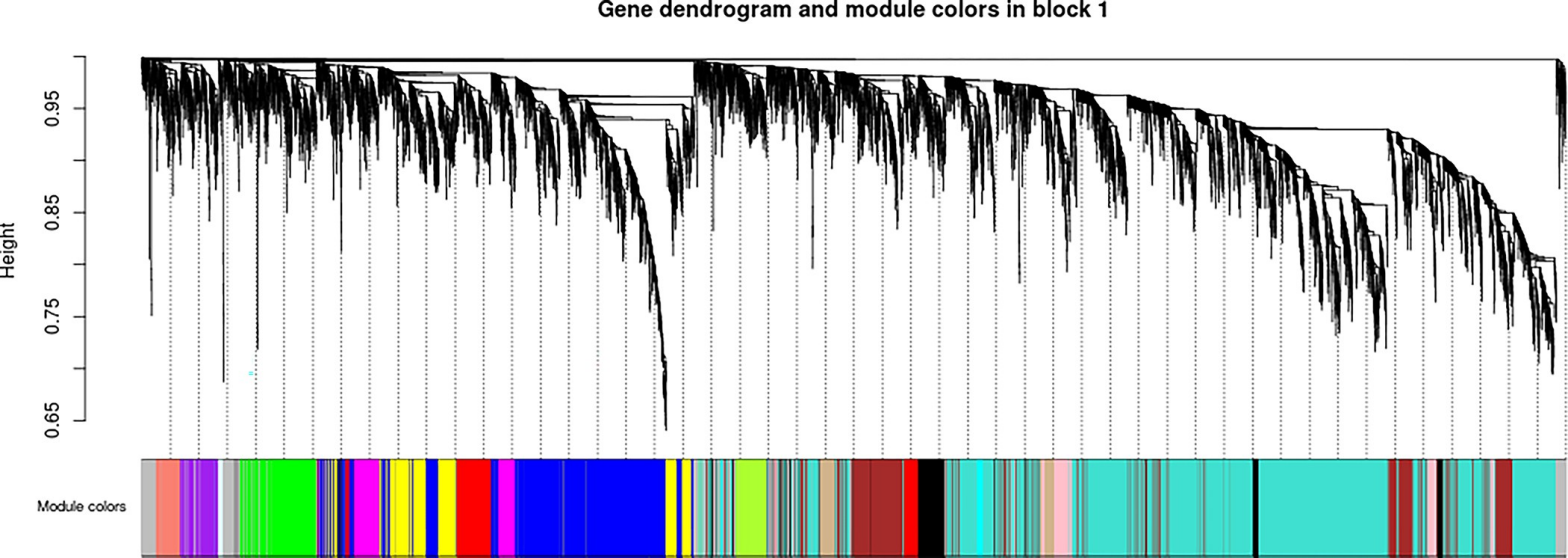
Hierarchical clustering tree of modules.

**Fig 8:**
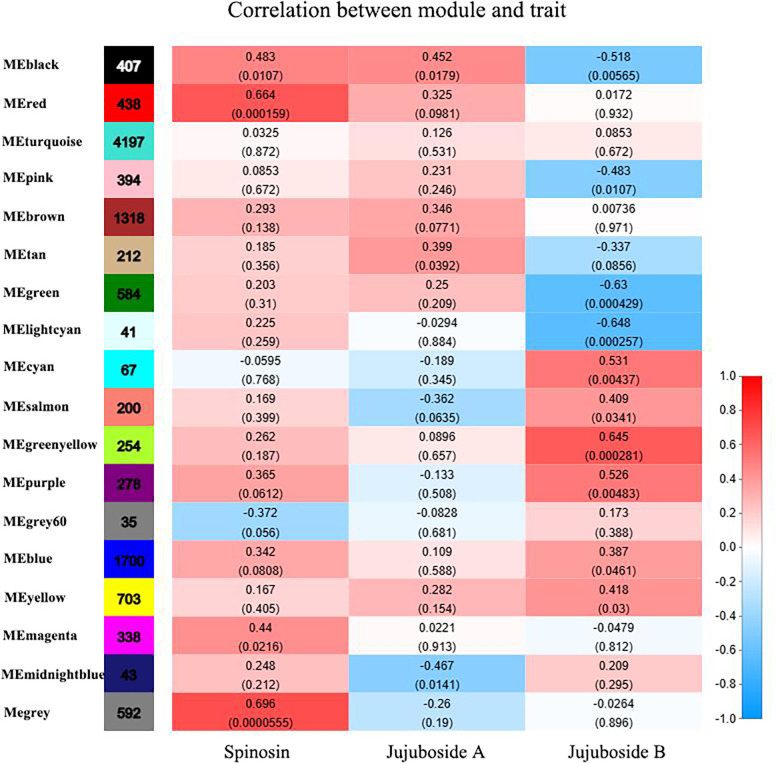
Correlation between modules and phenotypes of spinosin, jujuboside A, and jujuboside B.

WGCNA was conducted on 11,801 genes filtered from 27 SZS samples. The gene tree clustering diagram is displayed in Fig 7, where each branch represents a gene and each color represents a module. Eighteen modules were identified. Correlation analysis was then performed between the genes within these modules and the phenotypes, specifically spinosin, jujuboside A, and jujuboside B content, as illustrated in Fig 8. Among these modules, MEgray exhibited the strongest correlation with spinosin content, with a correlation coefficient (r) of 0.696 and a *P*-value of 5×10^−5^. The MEblack module showed a higher correlation with jujuboside A (r = 0.452, *P* = 0.0179), whereas the MEgreenyellow module demonstrated a higher correlation with jujuboside B (r = 0.645, *P* = 2.8×10^−3^).

#### Identification of core genes in modules

**Fig 9:**
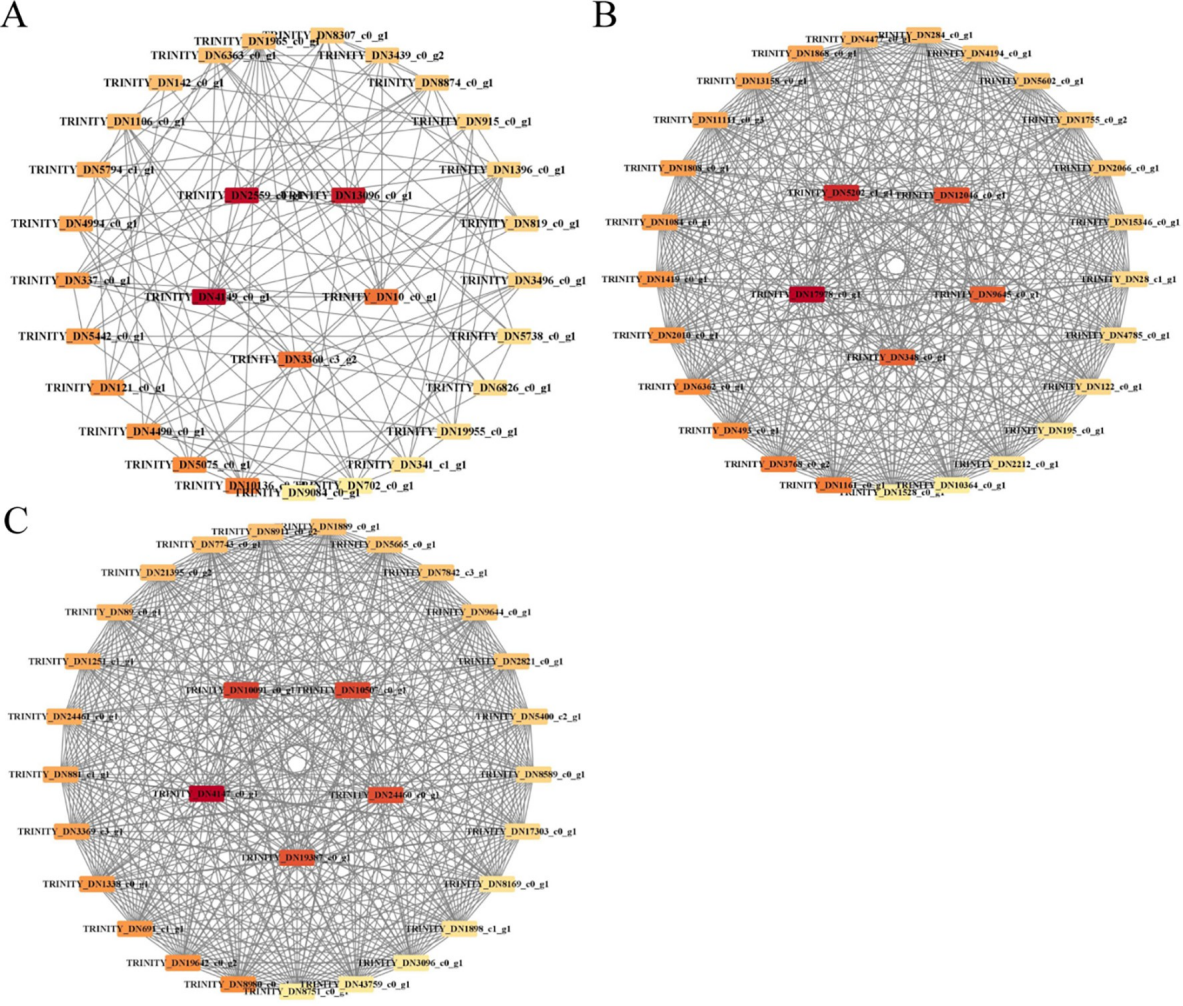
Visual analysis of modular genes. (A) Module MEgrey; (B) Module MEblack; (C) Module MEgreenyellow.

The Cytoscape software was used to visually analyze the MEgrey, MEblack, and MEgreenyellow modules. Based on their degree values, the top five genes with high connectivity to spinosin, jujuboside A, and jujuboside B were selected as the core genes. In total, 15 core genes were identified, as shown in Fig 9. Red indicates the higher importance of the genes. Further details of the core genes are presented in Table 4. *IspD*, an important enzyme in the *MEP/DOXP* pathway and a precursor of terpenoid synthesis, promotes the synthesis of intermediate isopentylpyrophosphate (*IPP*) and dimethylallyl pyrophosphate (*DMAP*) and increases the accumulation of terpenoids. IspD is considered the gene responsible for the difference in medicinal ingredients between grafted and wild SZS.

**Table 4:** Information of the core genes.

NO.	Gene ID	Gene name	Abbreviation
1	TRINITY_DN4149_c0_g1	ubiquitin carboxyl-terminal hydrolase 27	UCHs
2	TRINITY_DN2559_c0_g1	telomere repeat-binding factor 2	TRF2
3	TRINITY_DN13096_c0_g1	ubiquitin-like-specific protease 2A	USP2a
4	TRINITY_DN3360_c3_g2	Hypothetical protein L484_020566	L484_020566
5	TRINITY_DN10_c0_g1	2-C-methyl-D-erythritol 4-phosphate cytidylyltransferase	IspD
6	TRINITY_DN17978_c0_g1	non-structural maintenance of chromosomes element 4 homolog A	NSMCE4A
7	TRINITY_DN5202_c1_g1	uncharacterized protein LOC107407447	LOC107407447
8	TRINITY_DN12046_c0_g1	uncharacterized protein At1g51745	At1g51745
9	TRINITY_DN348_c0_g1	valine—tRNA ligase	vals
10	TRINITY_DN9645_c0_g1	uncharacterized protein LOC107417557	LOC107417557
11	TRINITY_DN4147_c0_g1	protease Do	DEGP1
12	TRINITY_DN10091_c0_g1	DNA replication licensing factor MCM2	MCM2
13	TRINITY_DN10507_c0_g1	Heterogeneous nuclear ribonucleoprotein	hnRNPs
14	TRINITY_DN24460_c0_g1	SEC12-like protein 1	PHF1
15	TRINITY_DN19387_c0_g1	methionine—tRNA ligase	MSM

### q-RT PCR analysis

**Fig 10:**
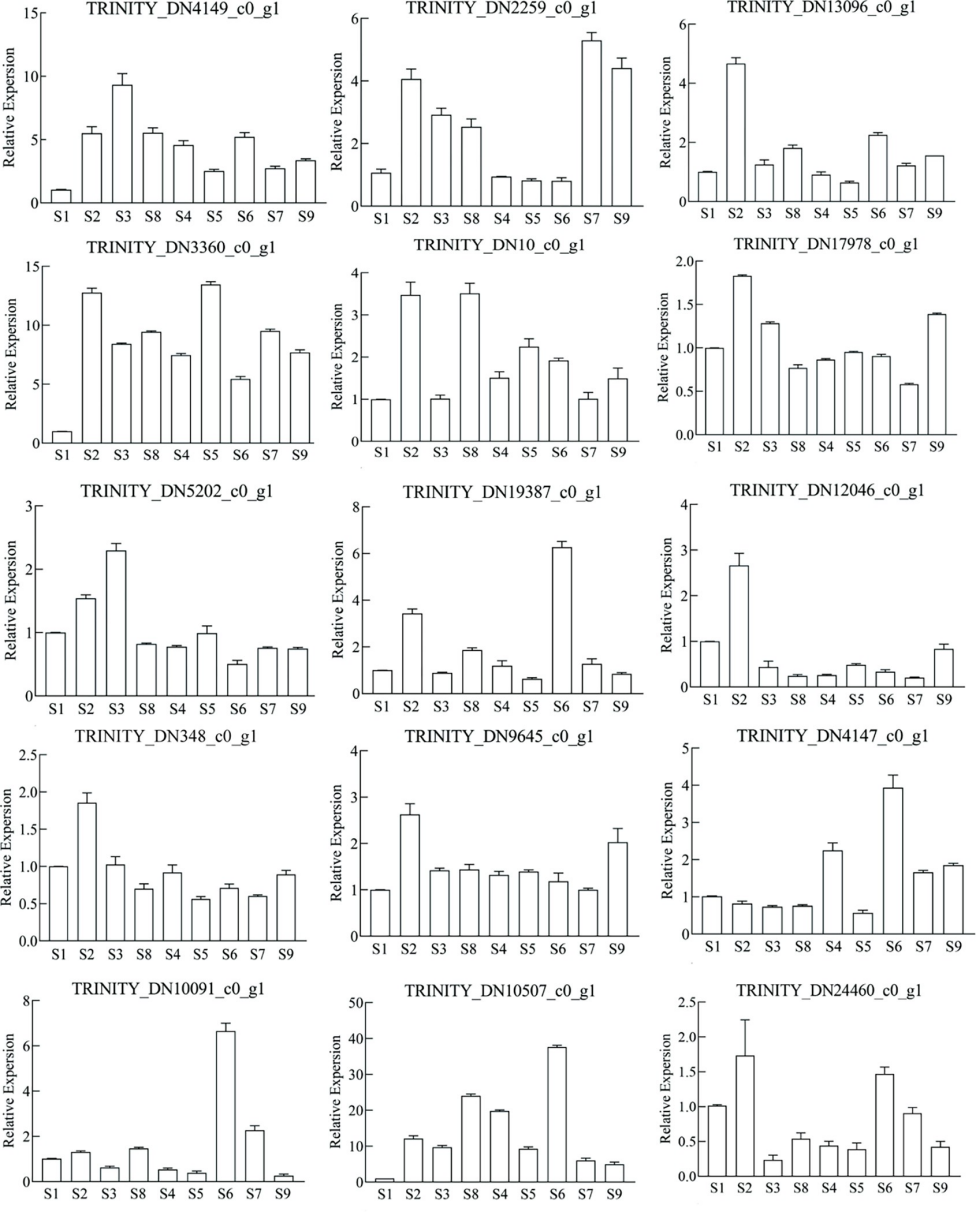
q-RT PCR analysis of 15 core genes related to the difference of medicinal components between grafted and wild Semen Ziziphi Spinosae (SZS).

A fluorescence quantitative PCR analysis was performed on the top 5 genes from the MEgrey, MEblack, and MEgreenyellow modules, totaling 15 core genes to verify the accuracy of the RNA-Seq data. The results presented in Fig 10 demonstrate that the expression patterns of these genes across different samples were largely consistent with the variations observed in gene expression abundance from transcriptome sequencing. This confirms the accuracy and reliability of the transcriptome data used in this study.

## Discussion

Identification methods for traditional Chinese medicine rely mainly on visual observations, taste, feel, smell, water and fire tests, and other means to determine authenticity [29]. Analysis of the characteristics of the nine samples revealed that the wild SZS date pits were oblong and reddish-brown. Samples S3, S7, S8, and S9 exhibited a shape similar to that of the wild SZS stones, and the S3 and S6 date pit colors were lighter than the wild SZS stones. Wild SZS are oval and purple-brown; most of the grafted SZS were purple-brown or purple-red, and a few were orange. These results indicated that grafting can induce phenotypic variations in SZS. Previous studies have shown that grafting can lead to phenotypic variations in plants [30–32], and our findings support this perspective.

DNA barcoding is an excellent method for species identification and is widely used in traditional Chinese medicine identification [25, 26, 33]. By investigating SZS characteristics, we showed that grafting affected the phenotypic variation of the SZS kernel. To determine whether the grafting affected the original properties of SZS, we established a DNA barcode for SZS. All 18 sample batches were identified as *Ziziphus jujuba* var. *spinosa*, indicating that grafting did not alter the original properties of SZS. The construction of the NJ tree revealed that the wild sample (S1) shared the same class as graft sample S6, which differed from the other grafted SZS samples. However, the average genetic distance was less than 0.005, indicating that grafting could introduce changes to the genetic material while maintaining a close genetic relationship.

To further investigate the differences in the medicinal components of SZS before and after grafting, the HPLC-ELSD method was used to determine the contents of spinosin, jujuboside A, and jujuboside B in both wild and grafted SZS. The contents of each component in the nine sample batches complied with the regulations outlined in the pharmacopeia, and the contents of each medicinal component in the grafted SZS were higher than in the wild SZS. Based on SA, HCA, PCA, and OPLS-DA, the components of grafted SZS from different rootstocks were consistent with those of wild SZS. The wild SZS exhibited a close distribution to most grafted SZS, while a few grafted SZS samples were dispersed. This suggests that the differences between the wild and grafted SZS were minimal. Wen and Wu also detected spinosin, jujuboside A, and jujuboside B in grafted SZS, which is consistent with our results [34, 35].

Flavonoids and saponins are the primary substances in SZS responsible for their sedative and hypnotic effects [36, 37]. To further understand the quality differences between grafted and wild SZS, up-regulated genes in flavonoid- and terpenoid-related pathways were screened for heatmap analysis. Ten genes in the flavonoid biosynthesis pathway and 11 in the saponin biosynthesis pathway were up-regulated in sample S8, indicating its superior quality. In the flavonoid biosynthesis pathway of sample S8, *C4H* catalyzed the production of coumaroyl-CoA, *CHS* facilitated chalcone formation, and naringenin is produced by isomerization of *CHI*. Dihydroflavonoids are crucial precursors of flavonoids [38–40]. Up-regulation of the aforementioned genes in the flavonoid biosynthesis pathway of sample S8 promoted the accumulation of dihydro flavonoids and facilitated the production of isoflavones, flavones, and dihydro flavanols under the catalysis of various enzymes, consequently increasing flavonoid content.

*HMGR* and *MVK* are vital enzymes in the mevaleric acid pathway (*MVA*), whereas MCS is a crucial enzyme in the *MEP/DOXP* pathway. These two pathways work in conjunction to generate IPP and its double-bonded isomer *DMAP* [41–43]. *IPP* and *DMAP* are catalyzed by enzymes such as *FPPS* and *SQE* to produce diverse terpenoids with varying molecular weights. Various triterpenoid saponins are formed via isomerization and methylation. The up-regulation of *HMGR*, *MVK*, *FPPS*, and *SQE* in sample S8 contributed to the accumulation of triterpenoid saponins. Studies have shown that grafting improves plant quality [44, 45]. Our study also showed that grafting improved the quality of SZS and explored the mechanism of quality differences through transcriptome analysis, providing a basis for further research on the quality improvement of SZS after grafting.

WGCNA was used to further analyze the core genes that influenced the differences in medicinal components between the grafted and wild SZS. Analysis of the core differential genes revealed that, except for *IspD*, which is a related enzyme in the biosynthetic pathway of the terpenoid skeleton, most genes, including *UCHs*, *vals*, *hnRNPs*, and *MSM*, were predominantly involved in primary metabolic processes. As a result, we speculated that grafting could impact primary metabolism and consequently affect the accumulation of secondary metabolites, such as spinosin, jujuboside A, and jujuboside B. This study provides data for further investigation of the mechanisms underlying the formation of quality differences in grafted SZS.

## Conclusions

In this study, DNA barcoding and HPLC-ELSD fingerprinting were used to identify grafted and wild SZS species, enabling the comparison of their genetic material and chemical components. This approach offers a novel perspective for the medicine quality evaluation technology. Transcriptome analysis was conducted to assess the expression of different genes involved in the flavonoid and saponin biosynthesis pathways in each sample. The core genes influencing the differences in medicinal components between the grafted and wild SZS were identified using WGCNA. The findings of this study provide a foundation for further exploration of the mechanisms influencing quality differences between wild and grafted SZS.

## Supporting information

S1 Table Chromatographic conditions. (DOC)

S2 Table Primer sequences for q-RT PCR. (DOC)

S3 Table Measurements of stones and kernel characters of sour jujube. (DOC)

S4 Table Sequence information and molecular identification of the nine SZS samples. (DOC)

S5 Table K2P genetic distance based on ITS2. (DOC)

S6 Table Linear relationship of components in SZS. (DOC)

S7 Table Contents of each component in SZS. (DOC)

S8 Table Similarity analysis of fingerprint of SZS. (DOC)

S1 Fig HPLC-ELSD spectrum of SZS. (A) Chromatogram of the standard. (B) Sample chromatogram. 1: Spinosin; 2: Jujuboside A; 3: Jujuboside B.
(TIF)

S2 Fig HPLC-ELSD fingerprint of SZS. (TIF)

S3 Fig Unigene length distribution. (TIF)

S4 Fig Analysis of differentially expressed genes between samples. (TIF)
