# Supplementary material for: DNA barcoding identification of grafted Semen Ziziphi Spinosae and transcriptome study of wild Semen Ziziphi Spinosae
Source: PLoS One. 2023 Dec 1;18(12):e0294944. doi: 10.1371/journal.pone.0294944 (PMC10691683; doi:10.1371/journal.pone.0294944)
Supplement: S1 Table — (DOC) [file pone.0294944.s001.doc]

S1 Table Chromatographic conditions

|  | Gradient elution | |
| --- | --- | --- |
|  | Acetonitrile (%) | Water (%) |
| 0 | 85 | 15 |
| 5 | 75 | 25 |
| 20 | 70 | 30 |
| 25 | 50 | 50 |
| 35 | 45 | 55 |
| 37 | 35 | 65 |
| 47 | 30 | 70 |
| 55 | 30 | 70 |
