# Supplementary material for: DNA barcoding identification of grafted Semen Ziziphi Spinosae and transcriptome study of wild Semen Ziziphi Spinosae
Source: PLoS One. 2023 Dec 1;18(12):e0294944. doi: 10.1371/journal.pone.0294944 (PMC10691683; doi:10.1371/journal.pone.0294944)
Supplement: S2 Table — (DOC) [file pone.0294944.s002.doc]

S2 Table Primer sequences for q-RT PCR

| Gene name | Primer sequence (5'→3') |
| --- | --- |
| Actin 8 | F: ATATGCTTGTCCATCTGGGAGG  R: ATTGACAAAACCACTGGCTGAG |
| TRINITY_DN4149_c0_g1 | F: TACCCGGATTACAAAACCTCG  R: CAGACCGTTCGCATTCCTCTA |
| TRINITY_DN2559_c0_g1 | F: GCTGCTGAGGACTTGGTTGAC  R: GCTCCATCGCAAGCATTACAC |
| TRINITY_DN13096_c0_g1 | F：CGCCACAAGGATGCTGACA  R: CGGGAAGCTCCTCCACAAA |
| TRINITY_DN3360_c3_g2 | F: AGCAAAAGGGAGAATATGGGG  R: AAGGGCTTCGCTGGAGAAAT |
| TRINITY_DN10_c0_g1 | F: TGGCTGGAGGGAAGGGTAA  R: TAAAAGGGTGATGGGGACGT |
| TRINITY_DN17978_c0_g1 | F: ATGGTACGGCCCGTCAAACGCGAAG  R: CTTCGCGTTTGACGGGCCGTACCAT |
| TRINITY_DN5202_c1_g1 | F: ATGGACGGGCTGAAATAAAAGT  R: GCTGTAAGAAACCTGCCCTGA |
| TRINITY_DN12046_c0_g1 | F: TCTGTGCAGGGAAGGAGACG  R: CATCCTACACCAATCCCCAAGT |
| TRINITY_DN348_c0_g1 | F: ATGCTTTACGATTCACGCTTTC  R: CATCGCTCTGACTAGGCAAATT |
| TRINITY_DN9645_c0_g1 | F: GCCCCTCACAAGTTCCTCCT  R: ATGCCCTCTATTCTTCCCATTT |
| TRINITY_DN4147_c0_g1 | F: TCTCCCTTTCTCCTCTGCTTTG  R: TGGCGAGTTCATCGGTCTG |
| TRINITY_DN10091_c0_g1 | F: CACGCTGCTCTGTTATTGCTG  R: CATAGGATGTCAAACCGGGAG |
| TRINITY_DN10507_c0_g1 | F:ATGGAAATGGAACCAGGCAAGCTAT  R: ATAGCTTGCCTGGTTCCATTTCCAT |
| TRINITY_DN24460_c0_g1 | F: TCTGTGATGTCCATCAGCAAGG  R: TGCAAGTACCCAGGTGTAGCC |
| TRINITY_DN19387_c0_g1 | F: TATAGCCATTGCATTGTCCCC  R: CCGCCCCACTTTGTCTCAT |
