# Supplementary material for: DNA barcoding identification of grafted Semen Ziziphi Spinosae and transcriptome study of wild Semen Ziziphi Spinosae
Source: PLoS One. 2023 Dec 1;18(12):e0294944. doi: 10.1371/journal.pone.0294944 (PMC10691683; doi:10.1371/journal.pone.0294944)
Supplement: S3 Table — (DOC) [file pone.0294944.s003.doc]

S3 Table Measurements of stones and kernel characters of sour jujube

| NO. | Semen ziziphi spinosae | | | Sour jujube date pit | | |
| --- | --- | --- | --- | --- | --- | --- |
|  | Longitudinal diameter (cm) | Transverse diameter (cm) | Thickness (cm) | Longitudinal diameter (cm) | Transverse diameter (cm) | Thickness (cm) |
| S1 | 0.63 | 0.46 | 0.27 | 1.28 | 0.67 | 0.63 |
| S2 | 0.50 | 0.46 | 0.24 | 1.44 | 0.98 | 1.03 |
| S3 | 0.64 | 0.51 | 0.31 | 1.10 | 0.72 | 0.66 |
| S4 | 0.67 | 0.51 | 0.26 | 1.11 | 0.74 | 0.75 |
| S5 | 0.75 | 0.53 | 0.28 | 1.63 | 0.80 | 0.78 |
| S6 | 0.98 | 0.65 | 0.34 | 1.25 | 0.70 | 0.58 |
| S7 | 0.75 | 0.62 | 0.31 | 1.15 | 0.83 | 0.84 |
| S8 | 0.81 | 0.65 | 0.28 | 1.18 | 0.72 | 0.76 |
| S9 | 0.63 | 0.57 | 0.28 | 0.97 | 0.72 | 0.75 |
