# Supplementary material for: DNA barcoding identification of grafted Semen Ziziphi Spinosae and transcriptome study of wild Semen Ziziphi Spinosae
Source: PLoS One. 2023 Dec 1;18(12):e0294944. doi: 10.1371/journal.pone.0294944 (PMC10691683; doi:10.1371/journal.pone.0294944)
Supplement: S4 Table — (DOC) [file pone.0294944.s004.doc]

S4 Table Sequence information and molecular identification of the nine SZS samples

| NO. | Length (bp) | GC (%) | Similarity (%) | Source |
| --- | --- | --- | --- | --- |
| S1 | 497 | 60.36 | [98.65](https://www.ncbi.nlm.nih.gov/nucleotide/MZ191009.1?report=genbank&log$=nucltop&blast_rank=1&RID=Y3F0D5RD016) | *Z. jujuba* var. *spinosa* |
| S2 | 492 | 60.97 | 99.78 | *Z. jujuba* var. *spinosa* |
| S3 | 493 | 60.46 | 98.65 | *Z. jujuba* var. *spinosa* |
| S4 | 497 | 60.56 | 98.37 | *Z. jujuba* var. *spinosa* |
| S5 | 460 | 62.17 | 100.00 | *Z. jujuba* var. *spinosa* |
| S6 | 518 | 60.04 | 99.54 | *Z. jujuba* var. *spinosa* |
| S7 | 489 | 60.94 | 97.49 | *Z. jujuba* var. *spinosa* |
| S8 | 494 | 60.53 | 98.65 | *Z. jujuba* var. *spinosa* |
| S9 | 515 | 60.58 | 99.79 | *Z. jujuba* var. *spinosa* |
