# Supplementary material for: DNA barcoding identification of grafted Semen Ziziphi Spinosae and transcriptome study of wild Semen Ziziphi Spinosae
Source: PLoS One. 2023 Dec 1;18(12):e0294944. doi: 10.1371/journal.pone.0294944 (PMC10691683; doi:10.1371/journal.pone.0294944)
Supplement: S5 Table — (DOC) [file pone.0294944.s005.doc]

S5 Table K2P genetic distance based on ITS2

|  | S1 | S2 | S3 | S4 | S5 | S6 | S7 | S8 | S9 |
| --- | --- | --- | --- | --- | --- | --- | --- | --- | --- |
| S1 | 1 |  |  |  |  |  |  |  |  |
| S2 | 0.002 | 1 |  |  |  |  |  |  |  |
| S3 | 0.002 | 0.004 | 1 |  |  |  |  |  |  |
| S4 | 0.000 | 0.002 | 0.002 | 1 |  |  |  |  |  |
| S5 | 0.002 | 0.000 | 0.004 | 0.002 | 1 |  |  |  |  |
| S6 | 0.002 | 0.000 | 0.004 | 0.002 | 0.000 | 1 |  |  |  |
| S7 | 0.036 | 0.038 | 0.038 | 0.036 | 0.038 | 0.038 | 1 |  |  |
| S8 | 0.002 | 0.000 | 0.004 | 0.002 | 0.000 | 0.000 | 0.038 | 1 |  |
| S9 | 0.002 | 0.000 | 0.004 | 0.002 | 0.000 | 0.000 | 0.038 | 0.000 | 1 |
