# Supplementary material for: DNA barcoding identification of grafted Semen Ziziphi Spinosae and transcriptome study of wild Semen Ziziphi Spinosae
Source: PLoS One. 2023 Dec 1;18(12):e0294944. doi: 10.1371/journal.pone.0294944 (PMC10691683; doi:10.1371/journal.pone.0294944)
Supplement: S6 Table — (DOC) [file pone.0294944.s006.doc]

S6 Table Linear relationship of components in SZS

| Medicinal ingredient | Linear relationship | *r*^2^ |
| --- | --- | --- |
| Spinosin | *Y*=10559*X*-309.88 | 0.9995 |
| Jujuboside A | *Y*=5113.4*X*-107.10 | 0.9998 |
| Jujuboside B | *Y*=3655.7*X*-44.719 | 0.9996 |
