# Supplementary material for: DNA barcoding identification of grafted Semen Ziziphi Spinosae and transcriptome study of wild Semen Ziziphi Spinosae
Source: PLoS One. 2023 Dec 1;18(12):e0294944. doi: 10.1371/journal.pone.0294944 (PMC10691683; doi:10.1371/journal.pone.0294944)
Supplement: S7 Table — (DOC) [file pone.0294944.s007.doc]

S7 Table Contents of each component in SZS

| Sample | Spinosin (mg/g) | Jujuboside A (mg/g) | Jujuboside B (mg/g) |
| --- | --- | --- | --- |
| S1 | 0.0913 | 0.0638 | 0.0191 |
| S2 | 0.1412 | 0.0758 | 0.0141 |
| S3 | 0.1319 | 0.0592 | 0.0137 |
| S4 | 0.1219 | 0.0386 | 0.0236 |
| S5 | 0.0820 | 0.0451 | 0.0139 |
| S6 | 0.0851 | 0.0657 | 0.0215 |
| S7 | 0.0930 | 0.0678 | 0.0185 |
| S8 | 0.0970 | 0.0758 | 0.0409 |
| S9 | 0.0903 | 0.0793 | 0.0130 |
