# Supplementary material for: DNA barcoding identification of grafted Semen Ziziphi Spinosae and transcriptome study of wild Semen Ziziphi Spinosae
Source: PLoS One. 2023 Dec 1;18(12):e0294944. doi: 10.1371/journal.pone.0294944 (PMC10691683; doi:10.1371/journal.pone.0294944)
Supplement: S8 Table — (DOC) [file pone.0294944.s008.doc]

S8 Table Similarity analysis of fingerprint of SZS

|  | S1 | S2 | S3 | S4 | S5 | S6 | S7 | S8 | S9 |
| --- | --- | --- | --- | --- | --- | --- | --- | --- | --- |
| S1 | 1 |  |  |  |  |  |  |  |  |
| S2 | 0.934 | 1 |  |  |  |  |  |  |  |
| S3 | 0.954 | 0.894 | 1 |  |  |  |  |  |  |
| S4 | 0.948 | 0.921 | 0.969 | 1 |  |  |  |  |  |
| S5 | 0.91 | 0.874 | 0.923 | 0.923 | 1 |  |  |  |  |
| S6 | 0.845 | 0.701 | 0.908 | 0.894 | 0.837 | 1 |  |  |  |
| S7 | 0.945 | 0.885 | 0.959 | 0.965 | 0.944 | 0.918 | 1 |  |  |
| S8 | 0.962 | 0.895 | 0.969 | 0.97 | 0.918 | 0.929 | 0.976 | 1 |  |
| S9 | 0.953 | 0.874 | 0.959 | 0.935 | 0.902 | 0.904 | 0.955 | 0.97 | 1 |
