# Supplementary figures and images for: DNA barcoding identification of grafted Semen Ziziphi Spinosae and transcriptome study of wild Semen Ziziphi Spinosae
Source: PLoS One. 2023 Dec 1;18(12):e0294944. doi: 10.1371/journal.pone.0294944 (PMC10691683; doi:10.1371/journal.pone.0294944)

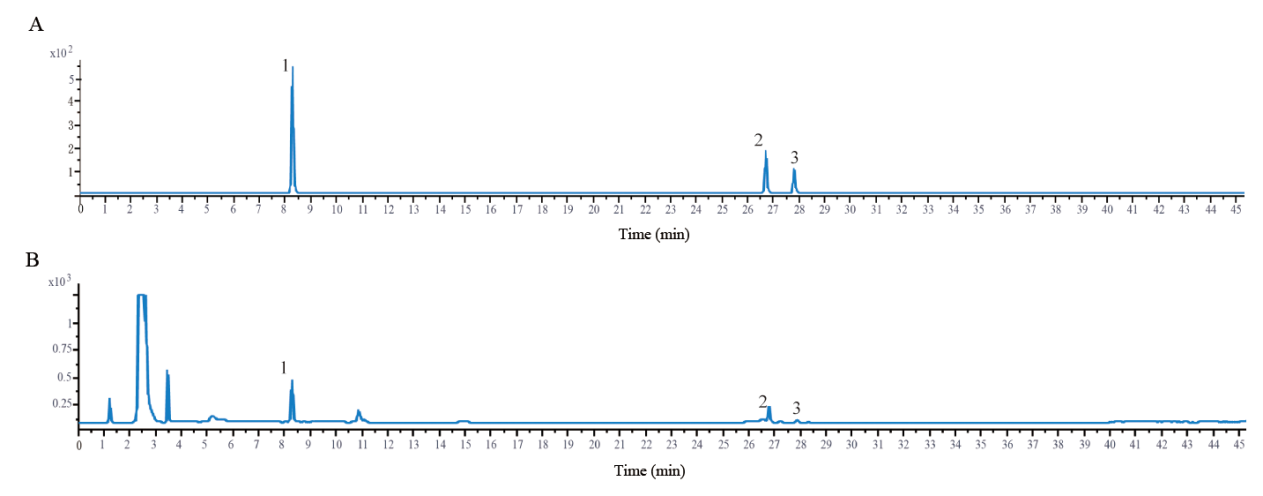

Supplement: S1 Fig — (A) Chromatogram of the standard. (B) Sample chromatogram. 1: Spinosin; 2: Jujuboside A; 3: Jujuboside B. (TIF) [file pone.0294944.s009.tif]

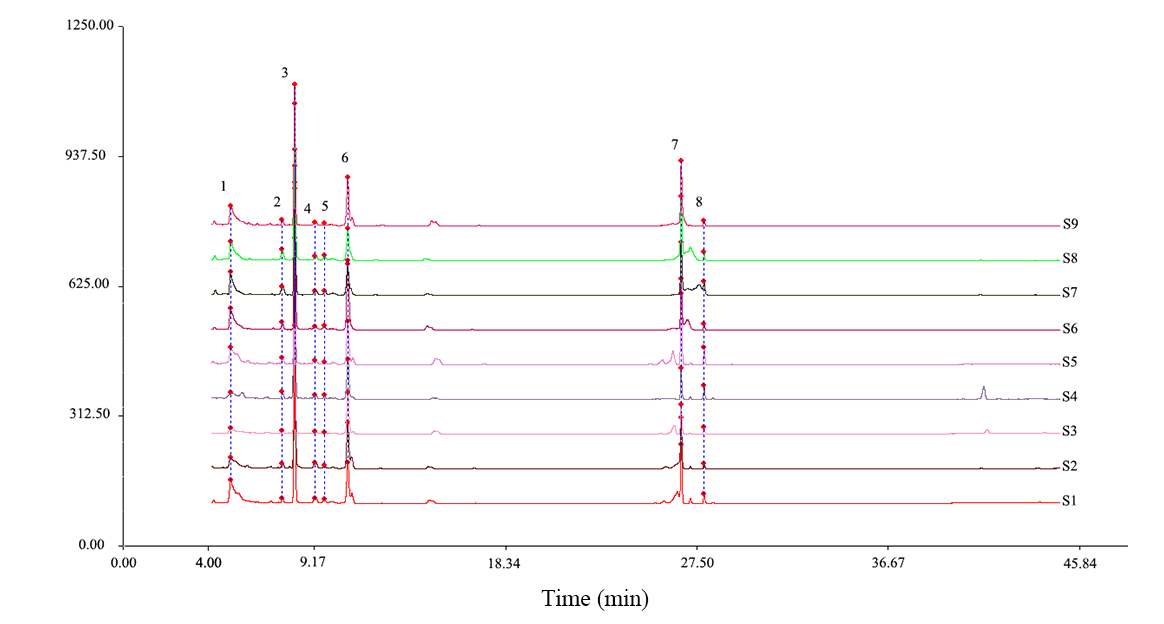

Supplement: S2 Fig — (TIF) [file pone.0294944.s010.tif]

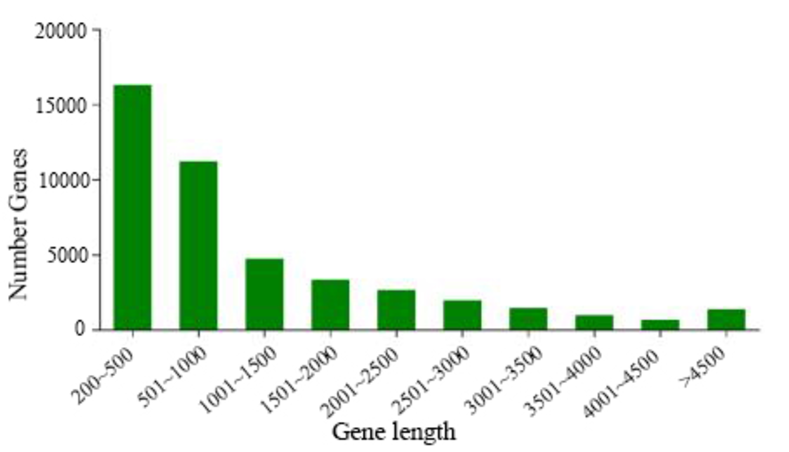

Supplement: S3 Fig — (TIF) [file pone.0294944.s011.tif]

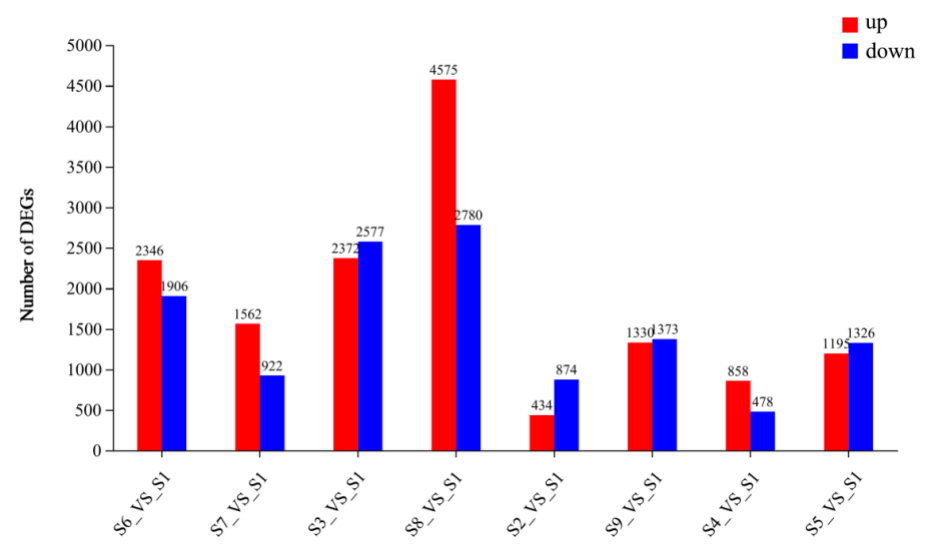

Supplement: S4 Fig — (TIF) [file pone.0294944.s012.tif]
